# Neurogenesis and Viral Infection

**DOI:** 10.3389/fimmu.2022.826091

**Published:** 2022-02-17

**Authors:** Amadi Ogonda Ihunwo, Jessica Perego, Gianvito Martino, Elisa Vicenzi, Paola Panina-Bordignon

**Affiliations:** ^1^ School of Anatomical Sciences, Faculty of Health Sciences, University of the Witwatersrand, Johannesburg, South Africa; ^2^ Neuroimmunology Unit, Division of Neuroscience, San Raffaele Vita-Salute University and IRCCS San Raffaele Hospital, Milan, Italy; ^3^ Viral Pathogenesis and Biosafety Unit, Division of Immunology, Transplantation and Infectious Disesases, IRCCS San Raffaele Hospital, Milan, Italy

**Keywords:** neural stem cells, neurogenesis, gliogenesis, ZIKV, SARS-CoV-2

## Abstract

Neural stem cells (NSCs) are multipotent stem cells that reside in the fetal and adult mammalian brain, which can self-renew and differentiate into neurons and supporting cells. Intrinsic and extrinsic cues, from cells in the local niche and from distant sites, stringently orchestrates the self-renewal and differentiation competence of NSCs. Ample evidence supports the important role of NSCs in neuroplasticity, aging, disease, and repair of the nervous system. Indeed, activation of NSCs or their transplantation into injured areas of the central nervous system can lead to regeneration in animal models. Viral invasion of NSCs can negatively affect neurogenesis and synaptogenesis, with consequent cell death, impairment of cell cycle progression, early differentiation, which cause neural progenitors depletion in the cortical layer of the brain. Herein, we will review the current understanding of Zika virus (ZIKV) infection of the fetal brain and the NSCs, which are the preferential population targeted by ZIKV. Furthermore, the potential neurotropic properties of severe acute respiratory syndrome coronavirus 2 (SARS-CoV-2), which may cause direct neurological damage, will be discussed.

## Neural Stem Cells

### Development of Neural Stem Cells

**Figure 1:**
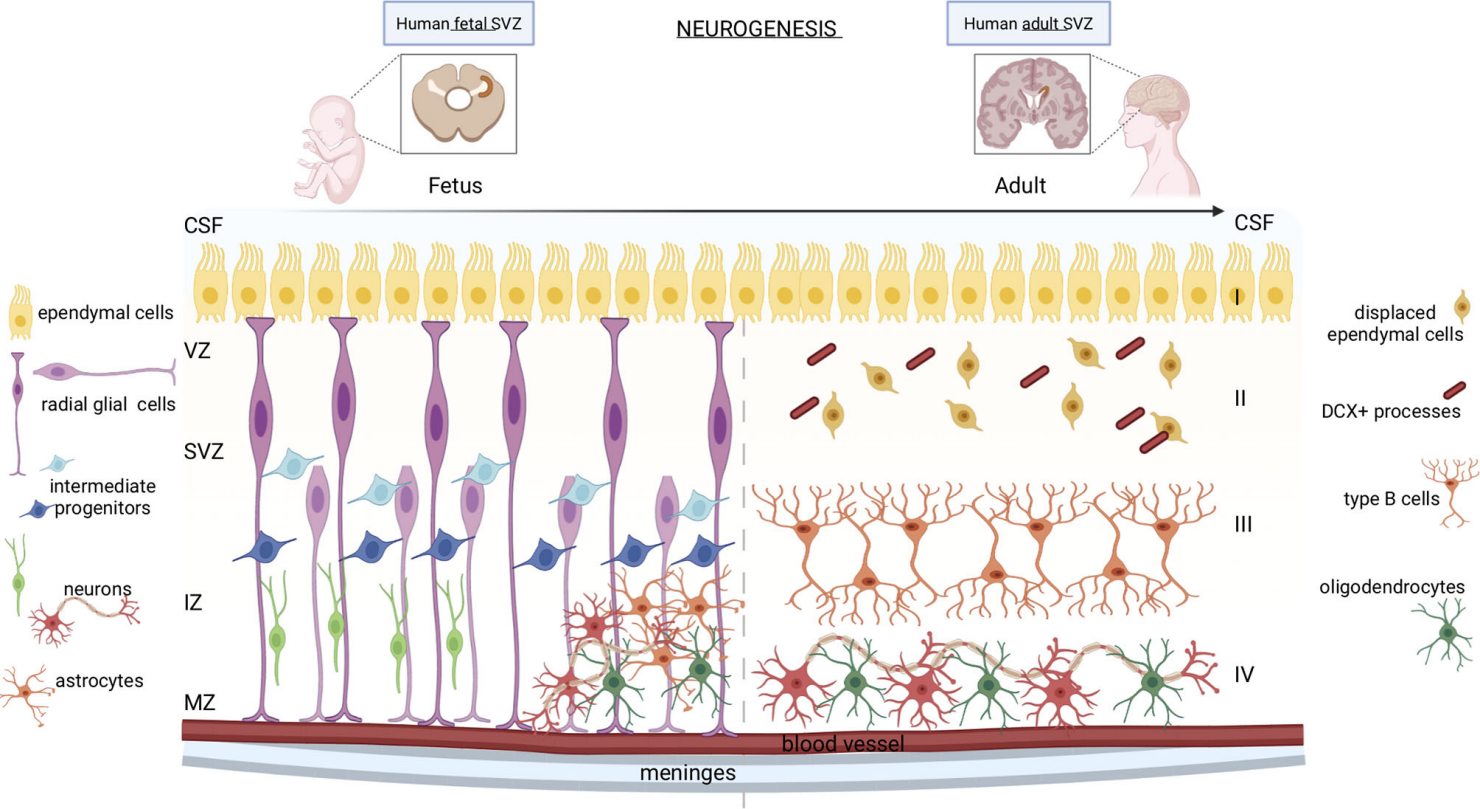
Neurogenesis in human fetal and adult SVZ. In the fetal brain, the neurogenic niche is organized into different strata: the ventricular zone (VZ), the subventricular zone (SVZ), the intermediate zone (IZ), and the marginal zone (MZ). Apical radial glial cells of the SVZ remain in contact with the ependymal cells and with the meninges and blood vessels, while the basal radial glial cells, which constitute the region of the SVZ called outer SVZ (oSVZ), lose contact with ventricles leading to a discontinuous scaffold of radial glial cells (RGCs). Apical and basal RGCs generate neurons through two mechanisms: either directly or indirectly through intermediate progenitor cells, which also originate astrocytes and oligodendrocytes. In the adult brain, the SVZ consists of four distinct stratified areas: layer I formed by ependymal cells in contact with the lumen of ventricles; the hypocellular gap (layer II), mainly formed by displaced ependymal cells and DCX+ astrocyte processes; layer III constituted by type B astrocytic cells; a transitional region to the brain parenchyma (layer IV) formed by mature neurons and oligodendrocytes.

Neural stem cells (NSCs) are multipotent stem cells present in the fetal and adult mammalian brain, which can self-renew and differentiate into the three main components of the central nervous system: neurons, astrocytes, and oligodendrocytes (1) (**Figure 1**).

During the early embryogenesis of mammals, the neural plate and neural tube comprise a single layer of proliferating neuroepithelial cells. Around gestational week (GW) 7-9, neuroepithelial cells line the inner part of the neural tube and, later, of the cerebrospinal fluid (CSF)-filled ventricles, named the subventricular zone (SVZ) (**Figure 1**, left panel). Neuroepithelial cells form a pseudostratified layer of mitotically active cells that rapidly amplify their pool before they differentiate into ventricular radial glial cells (RGCs) (2). RGCs are polarized cells in contact with the monolayer of ventricular ependymal cells on the apical side, and with the meninges and blood vessels on the basolateral side (3, 4). The ependymal cells establish a barrier and a transport system between the brain interstitial fluid and the CSF, which support neurogenesis regulation (5). Unlike the SVZ of other mammals, the human expanding SVZ, between GW 14 to 17, entails of a smaller inner SVZ (iSVZ) and an expanded outer SVZ (oSVZ) separated by a cell-poor region, the inner fiber layer (IFL) (6). The human oSVZ contains a new class of actively proliferating progenitors, the basal radial glial cells, that lost contact with ventricles from the apical surface, leading to a discontinuous RG scaffold (7). The basal RGCs initiate asymmetric cell divisions to generate neurons, but then quickly differentiate into intermediate progenitor cells (IPCs), a type of transit-amplifying cell, which further mature into neurons. This mechanism leads to the formation of a highly heterogeneous population of progenitor cells that generate diverse subtypes of differentiated neurons (8). After the neurogenic stages, the human RGCs become gliogenic, generating astrocytes or oligodendrocytes (4). RGCs are often referred to as neural stem cells (NSCs) since they can differentiate into neurons, astrocytes, and oligodendrocytes. The peculiar architecture of the human SVZ sustains the development of several neuronal and glial cell types in the complex cerebral cortex of primates (9).

In the mammalian adult brain (**Figure 1**, right panel), NSCs are present only in two niches, the ventricular-subventricular zone (V-SVZ) of the lateral ventricles, and the subgranular zone (SGZ) of the dentate gyrus (DG) in the hippocampus, which are dedicated to the generation of young neurons of the olfactory bulb (OB) and hippocampus, respectively (10). The SVZ organization of the human brain differs from that of well-studied rodents, which allowed the characterization of several functions of NSCs. Indeed, the human SVZ consists of four layers: cell bodies are accumulated in a ribbon (layer III) separated from the ependymal layer (layer I) by a gap that is largely devoid of cells (layer II), originated as a consequence of neuroblast depletion (11). The astrocytic ribbon (layer III) contains cell bodies of large astrocytes, a subset of which proliferate *in vivo* and show *in vitro* multipotency and self-renewal characteristics. Layer IV is a transitional region to the brain parenchyma. During fetal development, the proliferative activity within the SVZ progressively declines (12), but it remains active in neonates, along the wall of the lateral ventricle, generating diverse subtypes of neurons (13, 14).

Little is known about the precise role of neural stem cells in the adult human brain. Although the debate is still open, it has recently been reported that some degree of neurogenesis persists in adulthood, contradicting two decades of history stating that the human brain has no regenerative capabilities (15). In 1998, Eriksson and colleagues detected adult hippocampal neurogenesis in a post-mortem study of brains from neoplastic patients treated with bromodeoxyuridine (BrdU) for tumor-staging purposes. Proliferating cells (BrdU+) have been found in both the SVZ of the lateral ventricle and the subgranular zone of the dentate gyrus. In SGZ, some of these newly generated cells were observed to be capable to differentiate into neurons (16). Ernst and colleagues reported the presence of neuroblasts not only in SVZ but also in the adjacent striatum, suggesting that neuroblasts and new neurons in the adult human striatum derive from the SVZ (17). Hippocampal cell turnover during adult life was also confirmed by the quantification of integrated radiocarbon into DNA of replicated cells (18). Different studies proved that hippocampal neurogenesis persists throughout adult life (19–21) showing a lower age-associated decline in humans compared to mice (18). The preservation of hippocampal neurogenesis during evolution could be related to human cognitive adaptability. Interestingly, in patients with advanced Alzheimer’s disease, hippocampal neurogenesis has been described to drop sharply (19). In a small cohort of patients with amyotrophic lateral sclerosis (ALS), neural progenitor proliferation was increased in the SVZ and decreased in the SGZ (22) Methodological challenges, however, render studies about adult human neurogenesis of difficult interpretation, and contradictory results may depend on the use of diverse technologies (23). The development of new tools such as single-cell RNA sequencing, neuroimaging techniques, and the identification of novel reliable NSC markers will clarify the role that adult human neurogenesis plays in hippocampal function, neuroplasticity, and brain repair.

### Neurogenic and Non-Neurogenic Functions

The NSC functions have been extensively studied in mouse models in which, under physiological conditions, they can be divided into neurogenic and non-neurogenic activities.

In the SGZ of the hippocampus, new neurons are generated to regulate and refine the existing neuronal circuits. Indeed, hippocampal NSCs have been shown to have an important role in adult behavior and other learning-related tasks, as the preservation of spatial memory, memory acquisition and maintenance (24). The effects on neurogenesis have been extensively described in animal models. Mice in which the apoptosis-promoting gene *Bax* was conditionally ablated in NSCs to potentiate neurogenesis, showed an increased behavioral performance when tested with a specific cognitive task (25). On the contrary, decreased neurogenesis is associated with a prolonged hippocampus-dependent period of associative fear memory, likely aimed at preserving learning abilities by disposing of old memories (26). In the SVZ, immature neurons and NSC perform different tasks. Immature neurons tangentially migrate to three main areas: the olfactory bulbs (OBs) along the rostral migratory stream (RMS), the human prefrontal cortex along the medial migratory stream (MMS) (13), and the frontal lobe along the arc pathway (14). NSCs residing within the SVZ may contribute to the maintenance and reorganization of the central nervous system, to neurocognitive maturation and plasticity, although their functional role remains controversial (24).

Results from recent studies showed that, besides pure neurogenic functions, NSCs might play a comprehensive range of bystander, non-neurogenic activities to maintain brain homeostasis (27). NSCs produce and secrete an array of mediators that, in turn, regulate complex functions in the brain. For instance, neuroblasts derived both from the SVZ and SGZ can phagocytose apoptotic neuronal progenitors, an essential function in maintaining neurogenesis (28). Moreover, NSCs can curb microglial activation, proliferation, and phagocytosis by secreting factors like the vascular endothelial growth factor. Unchallenged microglia present in the adult SGZ maintain the homeostasis of the neurogenic cascade by removing apoptotic newly born cells by bilateral crosstalk between NSCs and microglia (29, 30). Furthermore, as demonstrated by Snyder and colleagues, neurogenesis-deficient mice mount a more severe response to acute stress, by showing increased food avoidance, behavioral despair in the forced swim test, and anhedonia in the sugar preference test. Thus, SGZ-derived newly generated neuroblasts seem to dynamically regulate stress responses by controlling the hypothalamic-pituitary-adrenal axis (31).

## Gliogenesis

**Figure 2:**
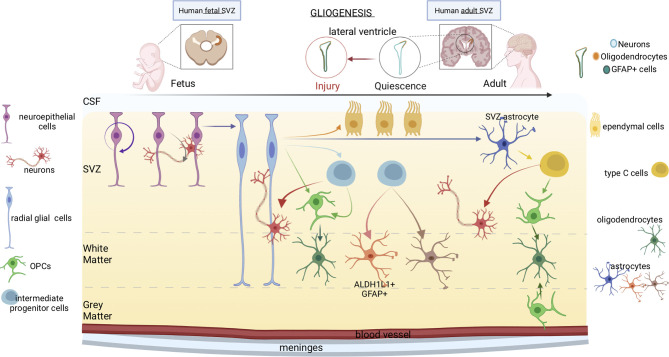
Gliogenesis in human fetal and adult brain. The progression from fetal to adult gliogenesis in the SVZ, which lines the lateral and septal sides of the lateral ventricle (LV), is shown. Arrows indicate cellular self-renewal or differentiation. Neuroepithelial stem cells, which can generate neurons, border the ventricles throughout the neuraxis at the stages of neural tube closure. At the beginning of the neurogenesis, neuroepithelial cells differentiate into radial glial cells. Radial glia produces intermediate progenitor cells and OPCs, which further differentiate respectively into neurons and oligodendrocytes. Radial glial cells have also the capacity to become astrocytes or produce intermediate progenitors. Lastly, intermediate progenitor cells can generate different subtypes of astrocytes. In adults, neural stem cells are neurogenic, yet, under some conditions like in response to injury, gliogenesis generate reactive astrocytes and oligodendrocyte-lineage cells. Oligodendrocytes are produced by either type C cells or OPCs located in the grey matter. Type C cells produce OPCs as well as neurons. The OPCs subsequently differentiate into oligodendrocytes.

Glia includes cells of ectodermal origin with diverse and dynamic functions - radial glia, astrocytes, oligodendrocyte progenitor cells (OPCs), oligodendrocytes - which orchestrate fundamental aspects of nervous system development and function (32). During brain development, distinct glia cells accomplish key tasks: neuronal birth, migration, axon specification, synaptogenesis, plasticity, homeostasis, constantly monitoring CNS structure and function (**Figure 2**). Transplantation experiments (33) showed that spinal cord progenitors that are restricted to glial lineage can recover neurogenic potential upon transplantation into the dentate gyrus, but not upon transplantation into the spinal cord or the non-neurogenic CA1 area of the hippocampus. Thus, adult glial progenitor cells are not lineage-restricted but can generate neurons upon exposure to appropriate environmental cues.

Astrocytes produce and secrete molecules that can drive the differentiation of adult neural stem/progenitor cells into neurons (33). Despite the adult hippocampus being composed greatly of neuroglia, which is four times more abundant than neurons (34), the lack of appropriate technical tools has delayed the study of the role of these supporting cells in adult neurogenesis. In recent days, the use of genetic tools and electron microscopy has started revealing that astrocytes and neural stem cells communicate with each other, both in physiological states and disease. It is now clear that astrocytes interact with neurons and other glial cells by secreting soluble mediators that act as gliotransmitters, neuromodulators, trophic factors, and hormones (35). Interestingly, some of these neuroactive molecules can exert either a driving or inhibitory role toward neurogenesis depending on the step in which they act. For instance, ATP, FGF2, and TSP1 have been found to stimulate adult NSCs (aNSC) proliferation (34). Also, neurogenesin-1, IL-1β, IL-6, and WNT3 have been shown to increase neuronal differentiation, while IGFBP6, enkephalin, and decorin reduced it. Neuronal maturation and synaptic integration are also boosted by D-serine. Moreover, Casse et al. (36) reported that astrocytes regulate the synaptic integration of new neurons by reducing connectivity and glutamate reuptake (34). It has also been documented by Toni et al. (37) that maturing neurons depend on pre-existing astrocytes to identify synaptic partners. In the dentate gyrus, the dendritic spines of new granule neurons generate synapses with axon terminals already on site.

Any disease or lesion of the nervous system that induces an immune activation promotes a reactive astrocyte phenotype, with increased expression of the glial fibrillary acidic protein (GFAP). More recently, transcriptomic analyses allowed a sharper distinction between diverse astrocytic subsets in pathological conditions. For instance, during neuroinflammation, the expression of genes involved in synaptic transmission and the release of neurotrophic factors are altered (38). As another example, in both patients and mouse models of Alzheimer’s disease, astrocytes rapidly respond to injury by becoming reactive and activating a series of molecular, cellular, and morphological changes (35, 36). Finally, cell surface expression of programmed cell death 1-ligand 1 (PD-L1) driven by the STAT3 pathway in reactive astrocytes is involved in the establishment of an immunosuppressive microenvironment in brain metastases (39).

NSCs also express astrocytic genes in response to the activation of diverse signaling pathways, triggered by morphogenic proteins (BMPs), which signals mainly through SMAD, leukemia inhibitory factor/ciliary neurotrophic factor (LIF/CNTF), which activates the JAK/STAT pathway, and the Notch pathway. *In vitro*, lipopolysaccharide (LPS), the classical inducer of neuroinflammation, stimulates microglia to release a NFκB-dependent secretome that includes interleukin 1 (IL-1), tumor necrosis factor TNF, and complement C1q (37).

Although the generation of astrocytes and their function in the adult brain are not yet well characterized, astroglia remains the predominant cell type of the neurogenic niche in terms of number of cells generated. In support of the important role of astrocytes in adult neurogenesis, Casse et al. (34) described how astrocytes can dysregulate adult neurogenesis leading to cognitive impairment in AD. Thus, a clear link exists between cognitive function and regulations of adult neurogenesis.

The process of differentiation along the oligodendroglial lineage is strictly coordinated by glia-glia and neuron-glia cross-talks at synaptic sites. Furthermore, according to Antel et al. (40), also immune-mediated mechanisms can contribute both positively and negatively to the generation and activation of OPCs. For instance, a subset of B lymphocytes, the B-1a cells, greatly contribute to OPC proliferation. B-1a cells can cross the blood-brain barrier in a CXCL13-CXCR5-dependent manner and are particularly abundant in the neonatal mouse brain. The fact that B-1a cells promote the proliferation of OPCs has been shown *in vitro* and further confirmed *in vivo* since the depletion of B-1a cells from the developing brain results in a reduction of both OPCs and mature oligodendrocytes. It has been demonstrated that B-1a cells secrete a soluble form of Fcα/μR, the receptor for the Fc region of IgM, which promotes OPCs proliferation and increases the axon myelination in the neonatal mouse brain (38). Altogether, these data demonstrate that B-1a cells infiltrating the brain may contribute to oligodendrogenesis and myelination by promoting OPC proliferation *via* activation of the IgM-Fcα/μR signaling pathway (38, 40).

## Neural Stem Cells As Viral Target

### Congenital Infections Affecting the Developing Fetal Neurodevelopment

TORCH infections are a group of congenital infections that can be transmitted from the mother to the fetus (41). The TORCH acronym refers to pathogens directly involved in the development of the congenital disease: **T**oxoplasma, **R**ubella, **C**ytomegalovirus, **H**erpes simplex 1 and 2, and **O**thers (Chlamydia, HIV, Coxsackievirus, Syphilis, Hepatitis B, Chickenpox, and ZIKV) (39, 40, 42–47). Although viral transmission during the third trimester of pregnancy has a reduced impact on the developing fetus, infection during the first trimester is extremely disruptive, with severe congenital neurological defects in the developing fetus, which include microcephaly, cognitive and intellectual disabilities, sensorineural hearing loss, and blindness. Evidence suggests that NSCs are directly affected by viral infections, which lead to developmental defects in the cerebral cortex mainly by interfering with their differentiation into mature neural cells (41). A summary of the main congenital syndromes associated with viral infections, is presented in **Table 1**.

**Table 1:** Main congenital syndromes associated with viral infections.

Pathogen	Genome	Family	Mother transmission route	Congenital syndrome	References
**Rubella Virus** **(RUBV)**	single- stranded RNA	Togaviridae	Aerosols	Microcephaly; diffuse and widely distributed calcification at basal ganglia; behavioral disorders; mental retardation.	(39, 42)
**Cytomegalovirus** **(CMV)**	double- stranded DNA	Herpesviridae	Blood transfusions, organ transplant and mucus exposure	Punctate and periventricular or cortical calcification; mental retardation; motor disabilities; hearing loss.	(40, 43)
**Varicella Zoster** **Virus** **(VZV)**	double- stranded DNA	Herpesviridae	Aerosols and contact with vesicular fluids	Microcephaly; ventriculomegaly; skin and extremities abnormalities.	(44)
**Herpes Simplex** **Virus (HSV) 1** **and 2**	double- stranded DNA	Herpesviridae	Sexual contact and ascending infection, perinatal infection	Skin and ocular abnormalities.	(45)
**Zika virus** **(ZIKV)**	single- stranded RNA	Flaviviridae	Mosquito bites, sexual	Microcephaly; ventriculomegaly; parenchymal or cerebellar calcification; arthrogryposis.	(46, 47)

Here we will focus on two viral outbreaks that created a substantial impact on public health: ZIKV and SARS-CoV-2. While ZIKV infection affects fetal neurodevelopment (48), SARS-COV-2 targets adult endogenous neurogenesis and affects homeostasis of neuronal circuits (49), while data on infected neonates are still scarce.

### The Case of ZIKV

**Figure 3:**
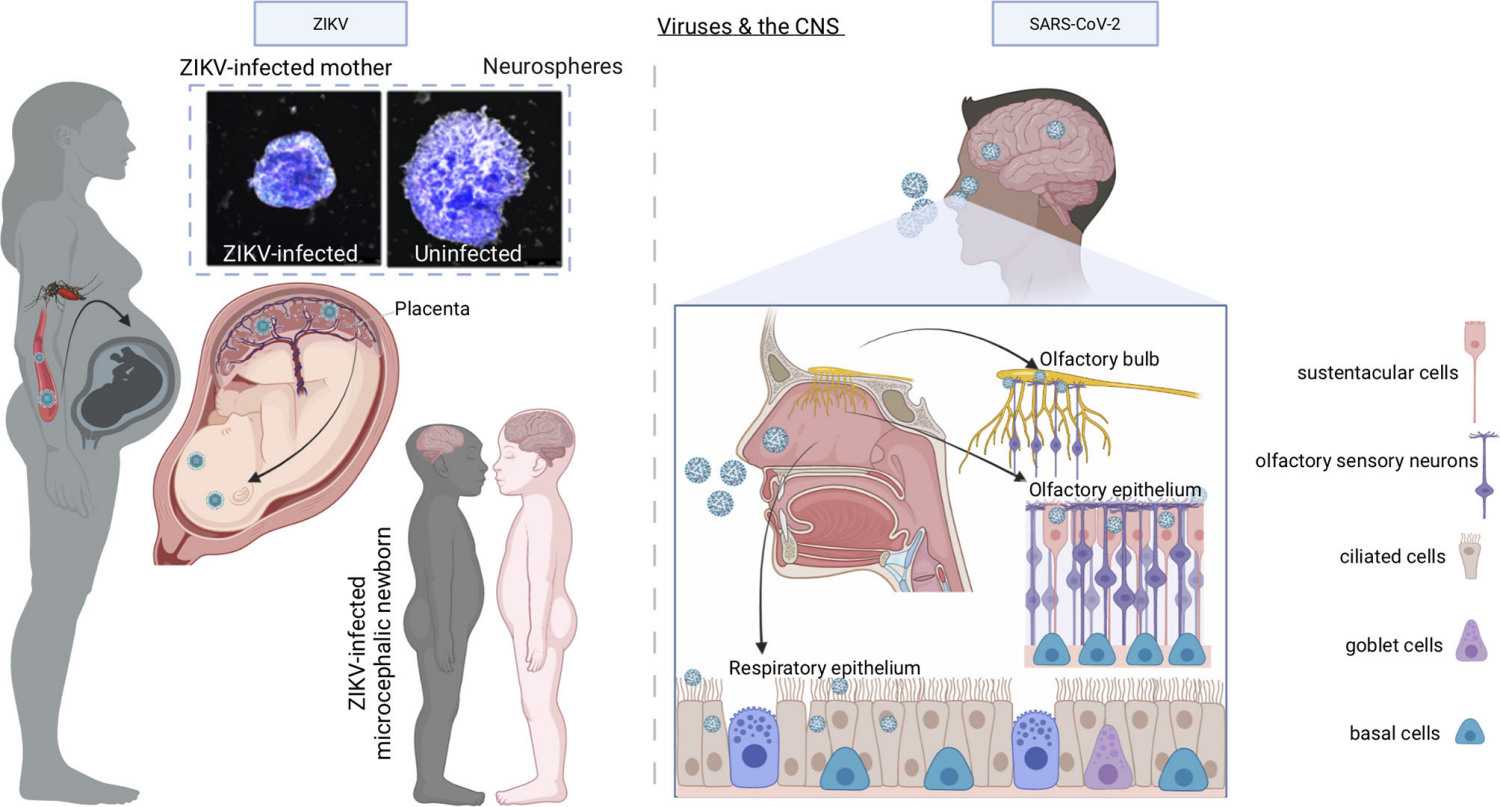
Models of ZIKV and SARS-CoV-2 infection of the CNS. When ZIKV is transmitted to a pregnant woman by bites of infected Aedes mosquitoes, although it causes mild or no symptoms in the mother, it enters the bloodstream, breaches the placental barrier, and infects the developing CNS of the fetus, with devastating consequences including microcephaly, decreased brain tissue, damage to the posterior segment of the eye, hypertonia, and arthrogryposis. *In vitro* experiments with human neurospheres confirm the direct effect of ZIKV on neural cells. Once SARS-CoV-2 is inhaled into the nasal cavity, it infects the sustentacular cells of the olfactory epithelium using ACE2 receptor and multiplies causing infection of the olfactory epithelium, the respiratory epithelium, and olfactory bulb through the numerous ACE2 receptors expressed by the cells of these tissues. From the olfactory bulb, the virus may travel to the CNS by retrograde axonal transport along olfactory sensory neurons.

ZIKV, a re-emerging arthropod-borne flavivirus, was firstly isolated from the blood of a febrile monkey in 1947 in the Zika forest of Uganda (50, 51). Through the 20^th^ century, few human ZIKV infections were reported, and these were recognized as mild non-life-threatening illnesses (52). Limited seroepidemiology surveys indicated that as many as 80% of infections were asymptomatic or subclinical (52). Therefore, little attention was paid to ZIKV up to the last decade when an outbreak of ZIKV infection occurred firstly in the Yap Island in the Federal State of Micronesia in April 2007 (52) and later in 2013 in French Polynesia when an increased incidence of Guillain-Barrè syndrome was reported to be associated with ZIKV infection (53). However, the rapid spread with millions of cases and the novel association of ZIKV with congenital microcephaly and the Guillain-Barrè syndrome changed the public health landscape such that the World Health Organization declared ZIKV pandemic a Public Health Emergency of International Concern (54) in 2016. Indeed, a seminal pathology study showed the presence of Zika virions and viral RNA in the microcephalic fetal brain with complete agyria and multiple microscopic abnormalities of an aborted fetus due to symptomatic maternal ZIKV infection acquired in Brazil (47). This study was followed up by more investigations, all confirming the pathologic spectrum of brain injury caused by ZIKV and lack of virus-induced cytopathic effects outside of the brain (55) (**Figure 3**, left panel).

ZIKV is a single-stranded RNA virus of the *Flaviviridae* family and is closely related to other members of this family, including Dengue, yellow fever, tick-borne encephalitis virus, West Nile, and Japanese encephalitis virus. ZIKV is commonly transmitted to humans by bites of infected *Aedes* mosquitoes (56). Differently from the closely related virus, cases of ZIKV sexual transmission have also been reported (57). Although the members of the *Flaviviridae* family mentioned above are neurotropic viruses that can cause severe illness with a significant possibility of permanent neurological damage or death (58), ZIKV causes a congenital ZIKV syndrome (CZS) only when the infection is acquired during the first and the beginning or whole second trimester of pregnancy (48, 59). Although some features of CZS are in common with other viral infections acquired during pregnancy as cytomegalovirus infection (60) and rubella (61), CZS is peculiar considering the severe microcephaly, decreased brain tissue, damage to the back of the eye, hypertonia, and arthrogryposis (https://www.cdc.gov/pregnancy/zika/testing-follow-up/zika-syndrome-birth-defects.html).

The finding of ZIKV in the amniotic fluid of pregnant women and the brain of microcephalic fetuses suggest a potential trans-placental infection route (47, 62). A potential source of the virus spreading to placental trophoblasts during the very early phases of pregnancy is represented by endometrial stromal cells, especially when decidualized by progesterone stimulation (63). ZIKV can reach and infect decidualized endometrial stromal cells *via* the uterine circulation or by sexual viral transmission.

#### *In Vitro* ZIKV Infection

The first evidence of the strong ZIKV tropism in NSCs came by comparing *in vitro* infection of iPSC derived NSCs with immature neurons, the last being less permissive to productive infection than NSCs (64). ZIKV envelope protein was detected in human iPS-derived NSCs 24 h after exposure to ZIKV, and infectious virus was detected in the cell culture supernatant 72 h post-infection, providing evidence of productive infection. Importantly, viral replication induced cell death and dysregulation of the cell cycle. To establish the connection between ZIKV infection and the malformations observed in fetal brains, Garcez et al. analyzed the impact of ZIKV infection in a 3D culture system of neurospheres derived from human iPSC (65). Viral particles were detected on the cell membrane, in mitochondria, and in intracellular vesicles of ZIKV-infected cells in the neurospheres. The presence of apoptotic nuclei, a hallmark of cell death, indicated that ZIKV was cytopathic for human NSCs, thus impairing the proper development of neurospheres.

To investigate how ZIKV infection affects brain development and causes microcephaly, 3D brain organoids derived from human embryonic stem cells can be used to recapitulate fetal brain development during the first trimester of pregnancy (66). Indeed, brain organoids self-organize and show regionalization, cortical differentiation, the presence of neuronal layers, and an outer RGC layer (66). ZIKV infection impaired the growth of human stem cell-derived organoids, with increased apoptosis, reduced proliferation and the ensuing decrease of neuronal cell-layer volume mirroring microcephaly (65, 67).

The analysis of the transcriptomic profile of human embryonic stem cell-derived organoids infected with a prototype strain of ZIKV showed that the innate immune receptor Toll-like-Receptor 3 (TLR3) was upregulated after ZIKV infection (68). Furthermore, TLR3 inhibition decreased the cytopathic effect of ZIKV infection. Pathway analysis of gene expression changes upon TLR3 activation identified several genes associated with neuronal development, indicating that ZIKV affects neurogenesis by interfering with a TLR3-regulated pathway. Thus, ZIKV-mediated activation of TLR3 severely affects neuronal cell fate, leading to an overall reduction of organoid volume mimicking a microcephalic phenotype (68).

#### Animal Models of ZIKV Pathology

Animal models of ZIKV infection have supported the characterization of ZIKV pathology. In this regard, direct evidence that ZIKV infection can cause microcephaly, with enlarged lateral ventricles and thinner cortical plates as compared to uninfected animals, was provided by Li and colleagues, who investigated ZIKV infection of the embryonic mouse brain, and its effects on brain development (69). Indeed, the Asian ZIKV strain, SZ01 replicates efficiently in embryonic mouse brain by directly targeting different neuronal lineages, including NSCs. ZIKV infected NSCs undergo cell-cycle arrest, apoptosis, and a differentiation blockage, ensuing cortical thinning and microcephaly. Gene expression analysis of infected brains showed the overexpression of flavivirus entry receptors and aberrant expression of genes related to immune responses and apoptosis.

The isolation of ZIKV from the amniotic fluids of pregnant women and the brain of microcephalic fetuses suggests a potential trans-placental infection route (47, 62). Decidualized endometrial stromal cells are a crucial target of ZIKV infection either *via* the uterine vasculature or by sexual transmission, thus likely representing a potential source of the virus spreading to placental trophoblasts during early pregnancy (63). The transplacental infection has been demonstrated in two mouse models of ZIKV infection during pregnancy: female mice lacking type I interferon signaling (*Ifnar1^−/−^*) crossed to wild type (WT) males, and pregnant WT females treated with an anti-ifnar-blocking antibody. In these models, ZIKV infected trophoblasts of the maternal and fetal placenta resulting in an intrauterine growth restriction (70). However, microcephaly, or deficiency of specific brain structures were not detected, possibly due to the different timing of brain development in mouse *vs*. human fetuses, as the development and maturation of the mouse brain includes a significant postnatal phase (71, 72).

In summary, ZIKV is a congenital infection that has serious consequences to the fetus and neonates and NSCs represent its preferred target. After infection, NSCs exit the cell cycle and die. Nevertheless, ZIKV has not been a major public health concern throughout the world since mid-2017 as after an estimation of 4,000 newborns with serious brain damage, the virus has disappeared from the Americas and the Caribbeans. However, an analysis of travelers who visited Cuba in 2017 or 2018 demonstrated ZIKV infection after their return to the United States and Europe (73). These results suggest that even during ZIKV waning infection, outbreaks were undetected until an immunologically naïve population of travelers became in contact with the virus. In the absence of an effective vaccine, travel surveillance is important, particularly for pregnant women.

### The Case of SARS-CoV-2

A novel severe respiratory disease emerged at the end of 2019 (coronavirus disease 2019, COVID-19) in Wuhan, China, and caused a still ongoing pandemic with more than 370 million people infected and 5 million deaths worldwide as of January 2022. COVID-19 is caused by a novel coronavirus called severe acute respiratory syndrome (SARS) CoV-2 (https://www.who.int/emergencies/diseases/novel-coronavirus-2019/technical-guidance/naming-the-coronavirus-disease-(covid-2019)-and-the-virus-that-causes-it) to distinguish it from SARS-CoV that emerged in the Guangdong province of China in 2003 and caused the severe clinical condition known as SARS (74). Like SARS-CoV, SARS-CoV-2 causes pneumonia with severe inflammation, which can progress to acute respiratory distress syndrome (ARDS) and death (75). COVID-19 can also be a multi-organ disease that may affect the brain (76–78) (**Figure 3**, right panel). Neurological manifestations including loss of smell and taste have been reported in concomitance with COVID-19 in approximately 27% of infected individuals (79) and can persist in subjects who have recovered from COVID-19 (80). However, it is unclear whether the sequela of neurological events depends on the direct infection of the neural tissue, or it is a consequence of the inflammation and activation of the coagulation cascade induced by the virus. In this regard, a recent report has demonstrated the presence of intact virions and SARS-CoV-2 subgenomic RNA (a surrogate of active viral replication) in the olfactory mucosa of a minority of autoptic specimens obtained from individuals who died of COVID-19 (81), suggesting that SARS-CoV-2 can access the central nervous system at the neural-mucosal interface of the olfactory mucosa *via* axonal transport. However, another study in which postmortem bedside collection of olfactory mucosa and whole olfactory bulbs was set up, failed to show the presence of SARS-CoV-2 in sensory neurons (82). These discrepancies might be explained by the difficulties to obtain samples of suitable quality from deceased individuals. Nevertheless, SARS-CoV-2 RNA was detected in the leptomeninges (82) suggesting that virions might have reached the cranial cavity either *via* migration through axonal transport or *via* cerebrospinal fluid and spillover from meningeal blood vessels. The analysis of single nucleus transcriptomes from both the frontal cortex and choroid plexus from autoptic samples of severe COVID-19, has shown major neuropathological phenotypes (49). SARS-CoV-2 was not detected in the brain although earlier neuroinvasion could not be excluded. These findings indicate that, in COVID-19 patients, cells of the blood-CSF barrier respond to inflammatory signals generated in the periphery by SARS-CoV-2 infection (83), allowing peripheral T cell infiltration (49).

To determine the potential SARS-CoV-2 neurotropism, iPSCs-derived neural cells have been used for *in vitro* infection with SARS-CoV-2 taking advantage of iPSC plasticity to be reprogrammed towards mature neuronal cells both in monolayer cells and structured organoids. To dissect the cellular effects of SARS-CoV-2 infection on the brain, McMahon et al. reported that glial cells and cells of the choroid plexus expressed the entry receptor for SARS-CoV-2 angiotensin-converting enzyme 2 (ACE2) but did not detect viral replication or cell death fragmentation (84). The recent development of cortical organoids containing pericyte-like cells (PLCs), allowed the researchers to demonstrate that PLCs can serve as SARS-CoV-2 ‘replication hubs’, sustaining viral invasion and spread to neighboring cells, including astrocytes (85). Indeed, a neuropathological study of post-mortem brain of COVID-19 patients found that astrocytes are the major site of SARS-CoV-2 infection and replication (86).

Strong evidence from both patients and experimental models indicate that human variants of SARS-CoV-2 could reach the CNS and target neurons, astrocytes, and microglia (87). The crosstalk between astrocytes and microglia plays a relevant role not only in the context of the local CNS inflammation but also in response to peripheral inflammation. In COVID-19 patients, neuroinflammation might arise and progress in response to the strong systemic cytokine storm observed in some patients, but also because of a CNS renin-angiotensin system dysregulation (87). Following SARS-CoV-2 infection of the brain, microglial cells get promptly activated, release an array of pro-inflammatory mediators, reactive oxygen species, and nitric oxide, recruit immune cells from the periphery and activate astrocytes (88–90).

SARS-CoV isolated from human specimens can infect C57/BL6 mice (91). Viral RNA was detected in the brain of infected mice up to 9 days post-intranasal infection while live virus could be isolated at later time point (9 to 15 days post-infection) (91). The virus was mainly localized in the hippocampus (91). Viral infection is associated with a strong neuroinflammatory response, which could either be induced by a direct viral infection of cells in the CNS or by the upregulation of peripheral cytokine levels. The activation of astrocytes and microglia in response to the elevation of peripheral cytokines is associated with a switch into a proinflammatory gene expression program, which could lead to increased blood-brain barrier permeability (87). Even if astrocytes and microglia may not be direct targets of viral infections, they can get activated in response to proinflammatory cues from endothelial cells, macrophages, and/or neurons, thus amplifying neuroinflammation. These data support the hypothesis that astroglia and microglia indeed play a relevant role in the development of the neurological symptoms observed in COVID-19 patients (87). However, the mechanisms by which the infected glia maintains the inflammatory reaction in the CNS remain to be addressed.

SARS-CoV-2 continuously evolves due to mutations that occur during replication of the genome. These mutations result in genetic variations of the circulating variants during the pandemic, which may spread more easily or show immune evasion and resistance to treatments. South Africa has witnessed the rapid emergence of SARS-CoV-2 variants. Some mutations in the C.1.2 lineage, a new lineage of the SARS-CoV-2 virus, have occured in other SARS-CoV-2 variants of concern. More data are being gathered to understand this new variant (National Institute for Communicable Diseases - NICD, 2021. Detection and frequency of the C.1.2 mutated SARS-CoV-2 lineage in South Africa.

https://www.nicd.ac.za/detection-and-frequency-of-the-c-1-2-mutated-sars-cov-2-lineage-in-south-africa/). This variant has not yet been investigated in terms of any effect on the brain and its cell types.

## Conclusions And Future Directions

### Use of 3D Models to Study Infection of Neural Progenitor Cells

Human brain organoids derived from iPSCs recapitulate the developmental process of the fetal human brain. They represent a physiologically relevant model to dissect mechanisms of neurodevelopment and study neurological diseases. Indeed, only the use of 3D models has revealed virus-specific and complex immune system strategies, emphasizing the power of brain organoids over 2D systems in modeling viral infections (85, 92).

Congenital viral infections caused by TORCH pathogens are a major cause of fetal brain malformation (93). However, the mechanisms by which distinct TORCH pathogens influence fetal neurodevelopment is still not known. Krenn et al. (92) have shown that brain organoid modeling of ZIKV and herpes simplex virus (HSV-1) infections reveal distinct virus-specific responses causing microcephaly. Both viruses efficiently replicate in early-stage brain organoids and reduce their growth by inducing cell death. However, transcriptional profiling shows that ZIKV and HSV-1 induce specific cellular responses. While HSV-1 activates non-neural developmental programs and impairs neuroepithelial identity, ZIKV infection induces the activation of antiviral and stress-related pathways without affecting the organoid cytoarchitecture. Furthermore, the two viruses display different sensitivities to type I interferons, although they both induce a weaker type I interferon response in 3D compared to 2D models.

SARS-CoV-2 has been linked to a wide variety of neurological conditions (94). The virus can infect the human CNS, either directly or indirectly *via* elusive mechanisms, leading to the inflammation of blood vessels and ensuing clotting, seizures, strokes, and hemorrhages. Recent studies showed that the virus entry receptor ACE2 is poorly expressed in neural cells, but highly expressed in brain pericytes, specialized cells that wrap around blood vessels and regulate immune cell entry to the CNS (95). Indeed, intranasal infection with SARS-CoV-2 induced a prompt hypoxic/ischemic-like pericyte response in the brain of transgenic mice expressing human ACE2 (95). Likewise, immunostaining of human brains demonstrated the presence of viral dsRNA in the vascular wall, perivascular inflammation, and a restricted loss of blood-brain barrier integrity (96). Since human brain organoids including only neural cells could not be infected with SARS-CoV-2, a human brain 3D model including also pericytes has been developed and shown to support the entry and infection of SARS-CoV-2 (85). This improved 3D model identified ACE2-expressing pericytes as one possible route of virus entry into the brain. Thus, pericytes can serve as a hub for SARS-CoV-2 amplification and spreading to other types of brain cells.

### Antiviral Agents Protecting Neural Progenitor Cells

Heparin, a soluble derivative of heparan sulfate widely used as anticoagulant, has potentially attractive features including inhibition of binding and entry of the enveloped viruses, such as herpes simplex (HSV) (97, 98), human immunodeficiency (HIV) (99), SARS coronavirus (100), and influenza (H5N1) (101). The study of heparin effects on ZIKV infection of human NSCs showed that heparin fully prevented ZIKV-induced cell death, while minimally affecting viral replication (102). Moreover, the differentiation potential of NSCs into neuroglia was fully preserved upon heparin-treatment (103).

Indeed, heparin can be exploited as an antiviral agent offering a fast therapeutic option for present and future emerging viruses. In this regard, the activity of heparin against SARS-CoV-2 has been established using a few *in vitro* experimental models (104, 105). Importantly, heparin used in both therapeutic and prophylactic anticoagulant regimes reduced in-hospital mortality compared with untreated patients (106). As COVID-19 is a disease that continues to occur despite highly efficacious vaccines, several drugs, marketed for other therapeutic indications, have been re-purposed to treat COVID-19 patients, and antiviral strategies that include treatment with remdesivir or convalescent plasma have received emergency approval (107, 108). Despite promising results, the use of such treatments is limited, as they can only be delivered intravenously. Additional treatments are therefore required and, indeed, the first orally available antiviral drug against COVID-19, molnupiravir has been approved for use in the UK. There is, therefore, an urgent need to develop additional treatments to curtail morbidity and mortality caused by SARS-CoV-2.

## Author Contributions

All authors contributed to the preparation and revision of the manuscript. AI mainly contributed to the gliogenesis and viral infection sections. JP prepared the figures. GM mainly contributed to the neurogenesis section. EV mainly contributed to the viral infection of neural stem cells and novel therapies sections. PP-B contributed to the preparation and revision of the manuscript and the figures. All authors contributed to the article and approved the submitted version.

## Funding

This work was supported by Progressive MS Alliance (collaborative research network PA-1604-08492, BRAVEinMS), the Italian Multiple Sclerosis Foundation ((FISM) Project ‘Neural Stem Cells in MS’), FRRB (Project PLANGECELL), Italian Ministry of Health RF-2016-02364155, Italian Ministry of Health COVID-2020-12371617.

## Conflict of Interest

The authors declare that the research was conducted in the absence of any commercial or financial relationships that could be construed as a potential conflict of interest.

## Publisher’s Note

All claims expressed in this article are solely those of the authors and do not necessarily represent those of their affiliated organizations, or those of the publisher, the editors and the reviewers. Any product that may be evaluated in this article, or claim that may be made by its manufacturer, is not guaranteed or endorsed by the publisher.
